# CircZFYVE1 functions as a competitive endogenous RNA to enhance LSM14A-mediated antiviral defense against influenza A virus

**DOI:** 10.3389/fimmu.2026.1841925

**Published:** 2026-07-09

**Authors:** Bingchen Qiao, Yiqing Zheng, Xiaoting Zhang, Menglu Fan, Zhiyuan Liu, Linjuan Wu, Juan Su, Jihui Ping

**Affiliations:** Ministry of Education (MOE) Joint International Research Laboratory of Animal Health and Food Safety, Engineering Center of Animal Immunity of Jiangsu Province, College of Veterinary Medicine, Nanjing Agricultural University, Nanjing, China

**Keywords:** ceRNA, circRNA, circZFYVE1, influenza A virus, LSM14A

## Abstract

**Introduction:**

Circular RNAs (circRNAs) are covalently closed endogenous RNAs that regulate gene expression at the post transcriptional level and have been implicated in antiviral immunity. However, their functional roles and regulatory mechanisms during influenza A virus (IAV) infection remain incompletely defined.

**Methods:**

circZFYVE1 expression was profiled in A549 cells infected with the WSN strain of IAV using RT qPCR under time course and dose response conditions. The mechanistic function of circZFYVE1 was investigated through miRNA target prediction, luciferase reporter assays, and functional assays assessing its role as a competing endogenous RNA (ceRNA) and its impact on innate antiviral signaling.

**Results:**

circZFYVE1 expression was induced in a time and dose dependent manner following WSN infection. Mechanistically, circZFYVE1 acts as a ceRNA by sponging hsa miR 4435, thereby relieving miRNA mediated suppression of the processing body associated protein LSM14A and enhancing innate antiviral signaling during infection.

**Discussion:**

These findings define a circRNA–miRNA–mRNA regulatory axis that links IAV infection to innate antiviral responses, providing new insights into circRNA mediated host defenses. The circZFYVE1/LSM14A pathway represents a potential target for future studies aimed at modulating endogenous antiviral immunity.

## Graphical Abstract

**Figure:**
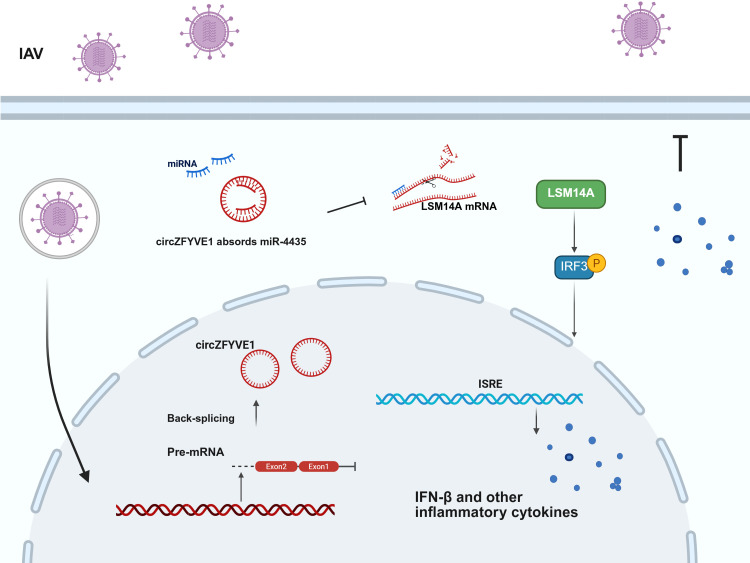
A novel circRNA-mediated regulatory axis controls antiviral immunity. Influenza A virus (IAV) infection induces expression of circZFYVE1, which acts as a competing endogenous RNA (ceRNA) by sponging hsa-miR-4435. This sponging activity prevents miR-4435 from suppressing LSM14A, a processing body-associated protein essential for type I interferon responses. Elevated LSM14A levels enhance innate antiviral signaling, inhibit IAV replication, and strengthen host defense, establishing circZFYVE1 as a positive regulator of antiviral immunity.The abstract should ideally be structured according to the IMRaD format (Introduction, Methods, Results and Discussion). Provide a structured abstract if possible. If your article has been copyedited by us, please provide the updated abstract based on this version.

## Introduction

Influenza viruses, members of the family Orthomyxoviridae, are segmented, single-stranded, negative-sense RNA viruses classified into four types: A, B, C, and D (1). Among these, influenza A virus (IAV) represents the most critical zoonotic pathogen. Due to its capacity for widespread transmission across diverse host species, IAV poses a persistent threat to global public health, causing seasonal epidemics and occasional pandemics that result in substantial morbidity, mortality, and economic losses (2–4). Despite the availability of vaccines and antivirals, the rapid mutation and genetic reassortment of IAV frequently result in vaccine mismatch and drug-resistant strains, highlighting the critical need to elucidate the complex host-pathogen interactions, particularly the regulatory networks governing host antiviral immunity.

Emerging evidence suggests that non-coding RNAs are pivotal players in diverse biological processes (5–7). Circular RNAs (circRNAs) are a unique class of regulatory molecules generated through the back-splicing of precursor messenger RNAs (pre-mRNAs), characterized by their covalently closed loop structures (8, 9). Although initially dismissed as aberrant splicing by-products, advances in high-throughput sequencing have established circRNAs as widespread and functionally critical components of the transcriptome (10). Recent studies have revealed that circular RNAs (circRNAs) play critical roles in various physiological and pathological processes through the regulation of epigenetic modifications, gene transcription, alternative splicing, RNA stability, and protein translation (11–14). Increasing evidence indicates that circRNAs also participate in innate and adaptive immunity. Mechanistically, circRNAs function primarily by acting as microRNA (miRNA) sponges to regulate immune gene expression, or by interacting with viral and host proteins to regulate viral replication and innate antiviral signaling pathways (15–17).

Increasing evidence suggests that circRNAs regulate viral infection and pathogenesis through distinct mechanisms. For instance, Circ-ATP5H facilitates hepatitis B virus replication via the miR-138-5p/TNFAIP3 axis (18), while the novel protein encoded by circMORC3 acts synergistically with its parental gene to dampen antiviral immunity (19).In the context of IAV infection, numerous host-derived circRNAs have been identified as key regulators of antiviral defense (20). Notably, Qu et al. identified an influenza virus-induced circular RNA termed AIVR, which antagonizes influenza virus replication by sponging miRNAs to enhance CREBBP expression, thereby promoting IFN-β production (21). In contrast to this sponge mechanism, circVAMP3 functions as a physical decoy for viral nucleoprotein (NP) and viral nonstructural protein 1 (NS1); this interaction disrupts viral ribonucleoprotein (vRNP) complex activity and counteracts NS1-mediated suppression of the RIG-I signaling pathway (16). Although high-throughput sequencing has identified hundreds of differentially expressed circRNAs during viral infections, the functional landscape of IAV-induced circRNAs remains largely unexplored.

LSM14A, also known as RAP55, is a multifunctional member of the LSm (Like-Sm) protein family that primarily localizes to processing bodies (P-bodies) and stress granules (22). It has been suggested to participate in shuttling mRNAs between P-bodies and stress granules. Beyond its classical roles in RNA processing and stability, LSM14A has been identified as a crucial sentinel in the innate immune system. As a cytosolic sensor, LSM14A has been reported to recognize viral nucleic acids, including viral RNA and DNA, thereby mediating IRF3 activation and IFN-β production in response to viral infection (23).In the context of RNA virus infection, LSM14A also functions as a restriction factor during IAV infection by sequestering viral NP into P-bodies, thereby impairing viral mRNA translation and suppressing viral protein synthesis (24). Despite its established importance, the post-transcriptional control of LSM14A expression remains obscure. Elucidating the regulation of LSM14A, particularly by non-coding RNAs, is essential to understand how host cells fine-tune antiviral responses to counteract viral evasion.

In this study, we identified circZFYVE1, a previously uncharacterized circRNA derived from the ZFYVE1 gene locus, as a novel positive regulator of innate antiviral immunity against IAV. We observed that circZFYVE1 is significantly upregulated upon IAV infection, enhances antiviral responses, and limits viral replication. Mechanistically, circZFYVE1 functions as a competing endogenous RNA (ceRNA) by sponging hsa-miR-4435, thereby relieving miRNA-mediated repression of LSM14A and amplifying downstream immune signaling. These findings define a novel circZFYVE1–miR-4435–LSM14A post-transcriptional regulatory axis that links IAV infection to the activation of host antiviral immunity. Importantly, elucidating this endogenous protective mechanism provides new insights into host-virus interactions and identifies the circZFYVE1/LSM14A pathway as a potential host-directed regulatory axis for future antiviral studies.

## Materials and methods

### Viruses and cells

The IAV strains used in this study included A/WSN/33 (H1N1) and A/Hong Kong/1/68 (H3N2), both of which were amplified in Madin-Darby canine kidney (MDCK) cells. The avian-origin A/chicken/Anhui/LH99/2017 (H9N2) strain was propagated via the allantoic cavity of 10-day-old specific pathogen-free (SPF) embryonated chicken eggs. Following harvest, viral stocks were aliquoted and stored at -80 °C. Viral infectivity was quantified using standard plaque assays on MDCK monolayers, and titers were expressed as plaque-forming units per milliliter (PFU/mL).

Human lung carcinoma epithelial cells (A549), MDCK cells, and human embryonic kidney 293T (HEK293T) cells were maintained in high-glucose Dulbecco’s Modified Eagle’s Medium (DMEM; Gibco, USA). The culture medium was enriched with 10% fetal bovine serum (FBS; Sbjbio, China) and a 1% antibiotic-antimycotic solution (penicillin/streptomycin; Gibco). All cell lines were incubated in a humidified atmosphere at 37 °C with a constant 5% CO_2_ supply.

### Antibodies and immunoblotting reagents

The following primary antibodies were used: anti-GAPDH rabbit polyclonal antibody (10494-1-AP; Proteintech), anti-IAV NP mouse monoclonal antibody (gift from Dr. Chengjun Li; Harbin Veterinary Research Institute, CAAS), anti-FLAG (SLAB0102; Smartlife sciences, China); Secondary antibodies conjugated with horseradish peroxidase (HRP) were used to visualize protein bands via an enhanced chemiluminescence (ECL) detection system.

### Oligonucleotides and plasmids

The primers used in this study were synthesized by Sangon Biotech (Shanghai, China) and General Biol (Anhui, China). The small interfering RNAs (siRNAs), microRNA (miRNA) mimics, and inhibitors in this study were synthesized by GenePharma (Shanghai, China). The comprehensive sequences of all oligonucleotides are detailed in **Supplementary Table 1**.

To achieve ectopic expression of circZFYVE1, the full-length linear sequence, flanked by 200-bp upstream and downstream sequences, was cloned into a modified pcDNA3.1 vector. This construct incorporated the MLLT3 intron 4 fragment (chr9: 20414651–20415428) to facilitate efficient circularization as described previously (25). To validate the circularization of plasmid-derived circZFYVE1, total RNA was extracted from A549 cells transfected with the circZFYVE1 expression plasmid. RT-PCR using divergent primers detected the expected circZFYVE1-specific back-spliced product in cDNA from transfected cells, and Sanger sequencing of the PCR product further confirmed the back-splice junction of circZFYVE1(**Supplementary Figure 1**). For the dual-luciferase reporter assays, the full-length sequence of circZFYVE1 or a 1,000-bp fragment of the LSM14A 3’ UTR, harboring either wild-type (WT) or mutated (MUT) miR-4435 binding sites, was inserted into the pmirGLO vector (Promega, USA). Specifically, the MUT constructs contained localized nucleotide substitutions within the predicted seed regions. For the FLAG-tagged LSM14A-3’UTR reporter assay, the LSM14A coding sequence together with its 3’ UTR (1,831 nt) was inserted into the pCMV-Flag vector. All constructs were verified by Sanger sequencing.

### RNase R resistance assay

To verify the circular stability of circZFYVE1, total RNA (10 μg) was subjected to digestion with 3 U/μg of RNase R (Beyotime, China) for 30 min at 37 °C, followed by heat inactivation at 70 °C for 10 min. Control samples were incubated under identical conditions without the enzyme. The treated RNA was purified via phenol-chloroform extraction and subsequently reverse-transcribed using random hexamers. The enrichment of circZFYVE1 and the depletion of linear transcripts were evaluated by RT-PCR using divergent and convergent primers, respectively, followed by 2% agarose gel electrophoresis to visualize the amplicons.

### Nucleocytoplasmic fractionation

To determine the subcellular distribution of circZFYVE1, A549 cells were challenged with the IAV WSN strain (MOI = 3). Cytoplasmic and nuclear RNA fractions were separated and purified using the Cytoplasmic & Nuclear RNA Purification Kit (Norgen Biotek, Canada) following the manufacturer’s instructions. The relative abundance of circZFYVE1 in each compartment was quantified via RT-qPCR. U6 small nuclear RNA (snRNA) was used as a nuclear control, whereas GAPDH mRNA was used as a cytoplasmic control.

### RNA quantification and expression analysis

The isolated RNA was subjected to reverse transcription and subsequent quantitative analysis. For relative quantification, cDNA was synthesized using HiScript II Q-RT SuperMix (Vazyme, China), which incorporates a genomic DNA (gDNA) removal step. Real-time PCR was performed employing the AceQ qPCR SYBR Green Master Mix (Vazyme) on a Roche LightCycler 96 platform. The thermal cycling conditions consisted of an initial denaturation at 95 °C for 5 min, followed by 40 cycles of 95 °C for 10 s and 60 °C for 30 s. Target gene expression was calculated using the 2^(-ΔΔCt) method/ the 2−ΔΔCt method (26), with GAPDH serving as the internal reference for normalization. The sequences for all specific primers utilized in this study are detailed in **Supplementary Table 1**.

### Transfection and viral infection

Oligonucleotides, including miR-4435 mimics/inhibitors, circZFYVE1-targeted siRNAs, and their respective negative controls, were obtained from GenePharma (Shanghai, China). For functional assays, cells were seeded in 12-well plates and transfected with 1 μg of expression plasmids or 80 nM of oligonucleotides using Lipofectamine 2000 (Invitrogen, USA) per well. For dual-luciferase reporter assays, HEK293T cells in 24-well plates were co-transfected with 100 ng of pmirGLO reporter vectors (WT or MUT) and 80 nM of miRNA mimics/inhibitors. Cells were lysed 24–48 hours post-transfection for subsequent luciferase activity measurement.

For infection, cells were washed with PBS and inoculated with virus diluted in serum-free DMEM at the indicated MOI for 1 h at 37 °C. The inoculum was then removed, and cells were maintained in infection medium containing TPCK-treated trypsin where appropriate. To evaluate viral replication, transfected cells were challenged with IAV at the specified MOI. Progeny virus production was quantified by harvesting supernatants at designated time points and determining titers via plaque assays on MDCK cells.

### Plaque assay

The infectious titers of influenza viruses were determined by plaque assays according to the procedures described previously (27). Briefly, viral samples were subjected to serial 10-fold dilutions and adsorbed onto MDCK cells for 60 min at 37 °C. Following inoculum removal, the monolayers were overlaid with DMEM supplemented with 1% SeaPlaque agarose (Lonza) and 1 μg/ml TPCK-treated trypsin. The plates were incubated at 37 °C for 48 hours. Plaque formation was visualized and counted to calculate the viral titers, expressed as plaque-forming units per milliliter (PFU/mL).

### Western blotting

Total cellular proteins were extracted using RIPA lysis buffer, resolved via SDS-PAGE, and electro-transferred onto nitrocellulose membranes (GE Amersham). Following a 1-hour blocking step with 5% non-fat milk at room temperature, the membranes were incubated with specific primary antibodies at 4 °C overnight. Subsequently, the blots were hybridized with corresponding HRP-conjugated secondary antibodies (Biodragon, China). Protein bands were visualized using enhanced chemiluminescence (ECL) detection reagents (Vazyme) and quantified with an Amersham Imager 600 analyzer (GE Healthcare).

### Luciferase reporter assays

Promoter Activity Analysis: To evaluate IFN-β and ISRE promoter activation, HEK-293T cells were seeded in 24-well plates and co-transfected with pGL-IFN-β or pGL-ISRE reporter plasmids, alongside the indicated expression vectors (pc3.1-circZFYVE1) or specific siRNAs. At 24 h post-transfection, cells were challenged with IAV (WSN, MOI = 1) for 12 h. Luciferase signals were quantified using the Steady-Glo Luciferase Assay System (Promega, USA) according to the optimized protocol.

miRNA Target Validation: To confirm the interaction between circZFYVE1 and miR-4435, cells were co-transfected with pmirGLO constructs (containing either WT or MUT sequences) and miRNA mimics using Lipofectamine 2000. After 36 h post-transfection, cells were harvested and processed with Passive Lysis Buffer (Promega). Both Firefly and Renilla luciferase activities were measured on a luminometer. The Firefly/Renilla ratio was calculated to determine relative bioluminescence, with each experiment performed in triplicate to ensure statistical reliability.

### RNA immunoprecipitation and RNA pull-down assay

To validate the interaction between circZFYVE1 and the RISC complex, Ago-RIP assays were conducted. Briefly, A549 cells (~2.0 × 10^7) were transfected with Flag-tagged Ago2 or the corresponding control vectors. At 48 h post-transfection, cells were harvested and lysed in specialized RIP buffer. The supernatants were then subjected to immunoprecipitation using magnetic beads conjugated with either anti-Flag or control IgG antibodies (Smartlife Sciences, China) at 4 °C overnight. The immunoprecipitated RNA was extracted from the beads and quantified via RT-qPCR to determine the relative enrichment of circZFYVE1.

To validate the physical interaction between miR-4435 and circZFYVE1, pull-down assays were performed using biotin-labeled miRNA mimics. Briefly, A549 cells were collected 48 h post-transfection and lysed in RIPA buffer supplemented with an RNase inhibitor (Beyotime, China). The resulting supernatants were incubated with M-280 streptavidin magnetic beads (Sigma-Aldrich), which had been pre-conjugated with biotinylated miR-4435, at 4 °C for 16 h. Following five washes with chilled lysis buffer to remove non-specific binding, the bead-bound RNA complexes were extracted using TRIzol reagent. The enrichment of circZFYVE1 in the pull-down fractions was quantified via RT-qPCR, with 10% of the total lysate reserved as the input control.

### Statistical analysis

Data are presented as mean ± standard deviation (SD) unless otherwise indicated. Statistical analyses were conducted using GraphPad Prism 8. Comparisons between two groups were performed using a two-tailed Student’s t-test, while comparisons among multiple groups were conducted using one-way or two-way ANOVA, as appropriate. Differences considered to be significant are indicated by asterisks as follows: *, P<0.05; **, P<0.01; ***, P<0.001. “ns” indicates no significance.

## Results

### Identification and expression characterization of circZFYVE1 in IAV-infected cells

**Figure 1:**
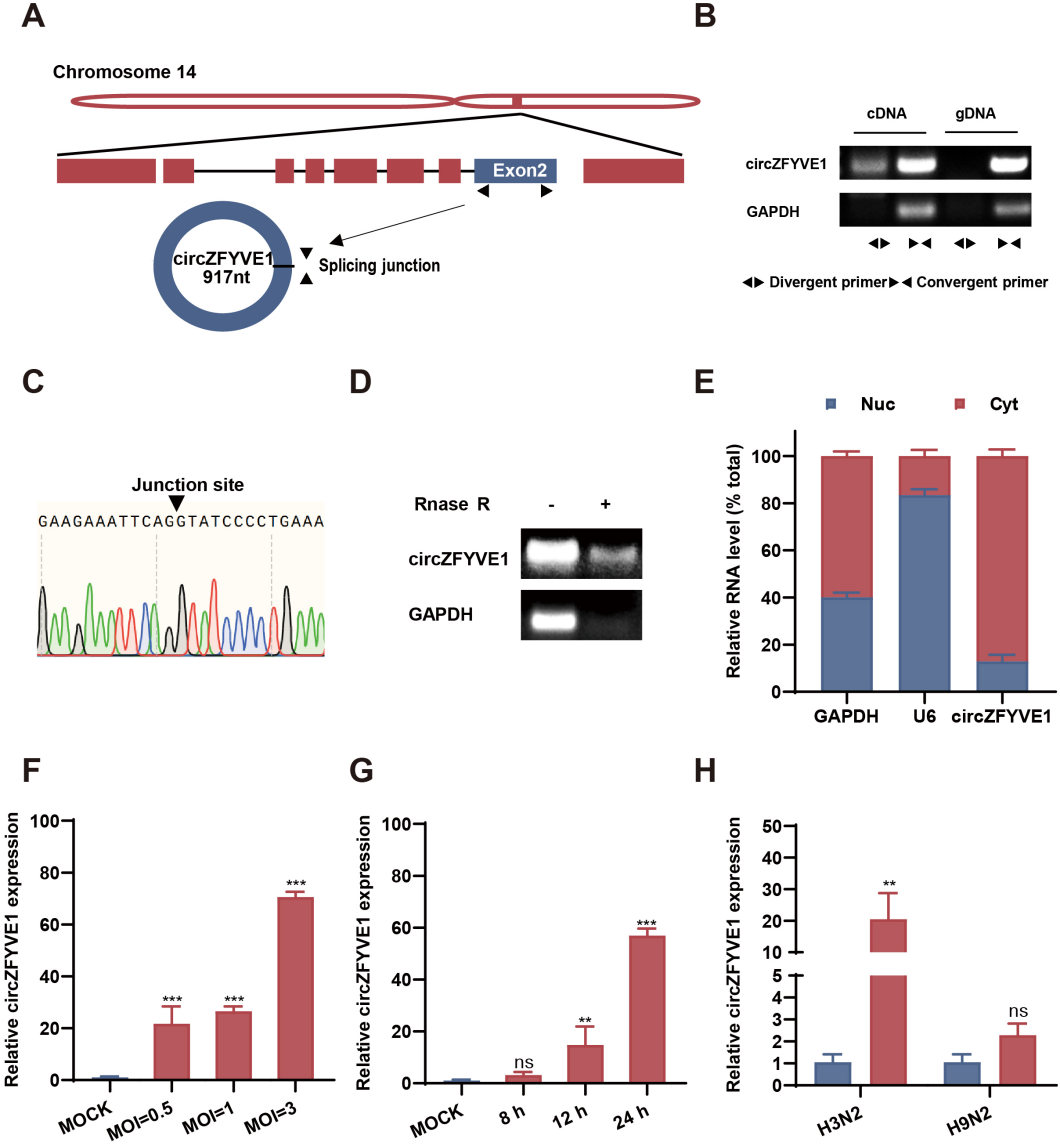
Identification and virus-inducible expression of circZFYVE1. **(A)** Schematic diagram of the ZFYVE1 genomic locus and the formation of circZFYVE1 through back-splicing of exon 2. The divergent primers used for specific amplification are indicated by black arrows. **(B)** Agarose gel electrophoresis of PCR products amplifying circZFYVE1. cDNA or genomic DNA (gDNA) from A549 cells was amplified using either divergent primers (specific for the back-splice junction) or convergent primers (specific for linear ZFYVE1 mRNA). **(C)** Sanger sequencing chromatogram of the PCR product from **(B)** using divergent primers. The black arrow indicates the back-splice junction site. **(D)** RNase R digestion assay. Total RNA from WSN-infected A549 cells was treated with or without RNase R. The relative levels of circZFYVE1 and linear GAPDH mRNA were analyzed by RT-PCR. **(E)** Subcellular distribution of circZFYVE1. qRT-PCR analysis of circZFYVE1, nuclear control U6, and cytoplasmic control GAPDH mRNA in fractionated nuclear (Nuc) and cytoplasmic (Cyt) RNA from A549 cells. Data show the percentage distribution (mean ± SD, n=3). **(F, G)** Expression profile of circZFYVE1 upon IAV (WSN) infection. **(F)** Dose-dependent induction. A549 cells were infected with WSN at the indicated MOI for 12 h. **(G)** Time-course induction. Cells were infected with WSN (MOI = 1) and harvested at the indicated time points. circZFYVE1 expression was determined by qRT-PCR and normalized to GAPDH. Data are mean ± SD (n=3). *p<0.05, **p<0.01, ***p<0.001 vs. 0 h or Mock (one-way ANOVA). **(H)** Induction of circZFYVE1 by different influenza A virus subtypes. A549 cells were mock-infected or infected with the indicated virus strains (MOI = 1, 24h). circZFYVE1 expression was analyzed by qRT-PCR and normalized to GAPDH. Data are mean ± SD (n=3). **p<0.01, ***p<0.001 vs. Mock (one-way ANOVA).

To investigate the potential involvement of circular RNAs in the host response to IAV infection, we performed RNA sequencing on A549 cells infected with the A/WSN/33 (H1N1) strain, as reported in a previous study (27). A circular RNA derived from exon 2 of the ZFYVE1 gene locus was identified and termed circZFYVE1. This circRNA is 917 nucleotides in length (**Figure 1A**). RT-PCR amplification using divergent primers confirmed the presence of the back-spliced junction, whereas convergent primers amplified the linear ZFYVE1 transcript (**Figure 1B**). Sanger sequencing of the divergent PCR product further verified the precise head-to-tail junction site (**Figure 1C**). The circular nature of circZFYVE1 was validated by its resistance to RNase R digestion, in contrast to the degradation of the linear GAPDH mRNA control (**Figure 1D**). Subcellular fractionation analysis revealed that circZFYVE1 was predominantly localized in the cytoplasm (**Figure 1E**), suggesting a potential role in post-transcriptional regulation.

Next, the expression dynamics of circZFYVE1 following WSN infection in A549 cells were examined. WSN infection led to a significant dose-dependent upregulation of circZFYVE1 in A549 cells (**Figure 1F**). A time-course experiment further showed that circZFYVE1 expression gradually increased after WSN infection, with significant induction beginning at 12 h post-infection (**Figure 1G**). To determine whether circZFYVE1 induction also occurs after infection with other IAV strains, we examined A/Hong Kong/1/68 (HK, H3N2) and H9N2. HK (H3N2) infection significantly increased circZFYVE1 expression, whereas H9N2 infection showed an increasing trend that did not reach statistical significance under the tested conditions (**Figure 1H**). These results identify circZFYVE1 as an IAV-responsive circular RNA whose induction varies among the tested IAV strains, suggesting its potential involvement in the host response to IAV infection.

### CircZFYVE1 restricts IAV replication in A549 cells

**Figure 2:**
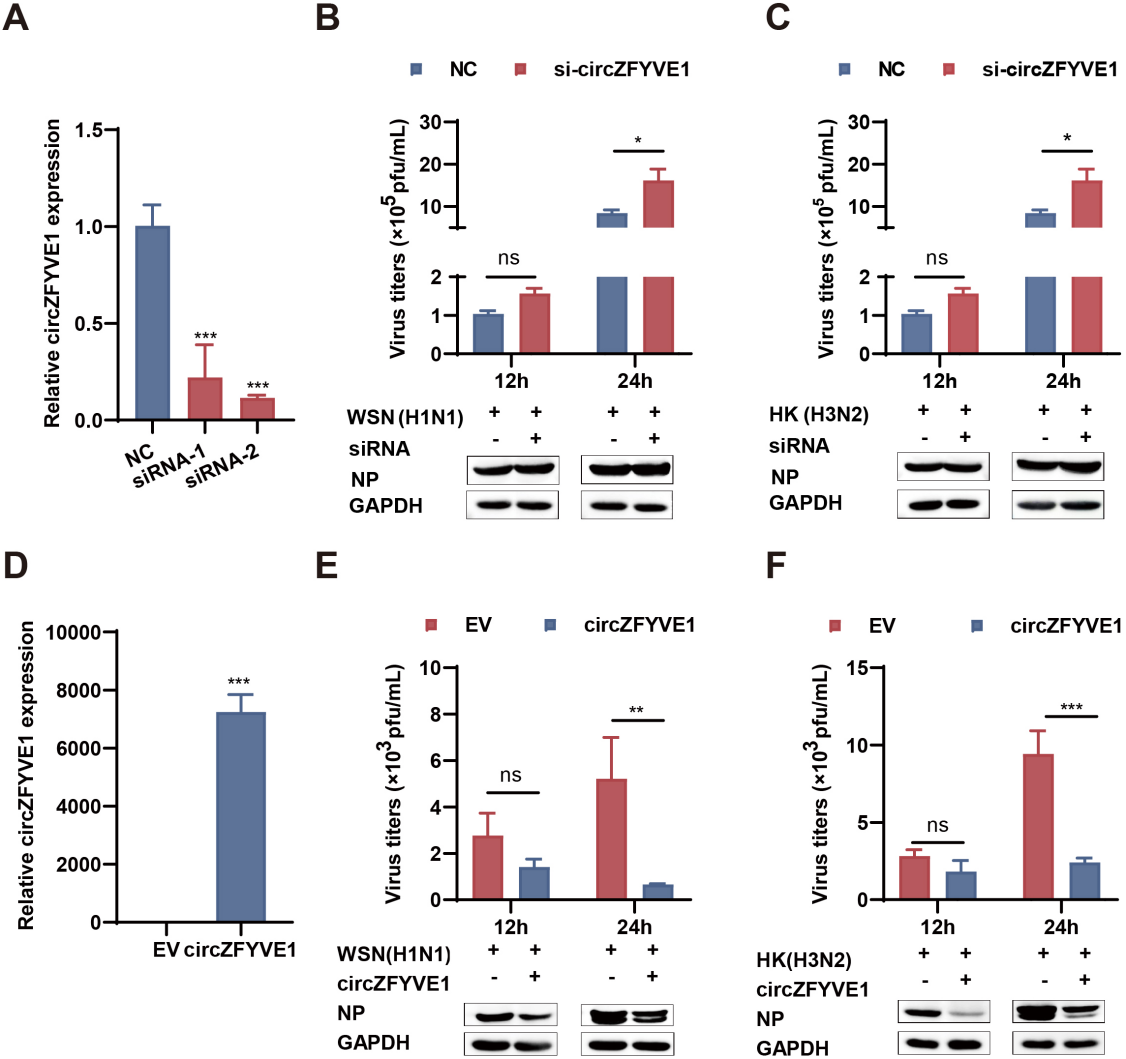
circZFYVE1 inhibits influenza A virus replication. **(A)** Specific knockdown of circZFYVE1. A549 cells were transfected with negative control siRNA (si-NC) or specific siRNAs against circZFYVE1. The expression level of circZFYVE1 was analyzed by qRT-PCR at 24 hours post-transfection. Data are normalized to si-NC and presented as mean ± SD (n=3). *p < 0.05, **p < 0.01, ***p < 0.001, ns, not significant (one-way ANOVA). **(B, C)** Knockdown of circZFYVE1 enhances IAV replication. Cells transfected with si-NC or si-circZFYVE1 were infected with **(B)** A/WSN/33 (H1N1) or **(C)** A/Hong Kong/1/68 (H3N2) virus at an MOI of 0.1. Supernatants were collected at 12 and 24 hpi, and virus titers were determined by plaque assay on MDCK cells. Data are mean ± SD (n=3). *p < 0.05, **p < 0.01, ***p < 0.001, ns, not significant (two-way ANOVA). **(D)** Efficient overexpression of circZFYVE1. Cells were transfected with empty vector (Vector) or the circZFYVE1 expression plasmid (circZFYVE1). The level of circZFYVE1 was quantified by qRT-PCR and normalized to GAPDH at 24 hours post-transfection. Data are mean ± SD (n=3). *p < 0.05, **p < 0.01, ***p < 0.001, ns, not significant (Student’s t-test). **(E, F)** Overexpression of circZFYVE1 inhibits IAV replication. Cells transfected with Vector or circZFYVE1 were infected with **(E)** WSN or **(F)** A/Hong Kong/1/68 (H3N2) virus at an MOI of 0.01. Virus titers in the supernatants at 12 and 24 hpi were measured. Data are mean ± SD (n=3). *p < 0.05, **p < 0.01, ***p < 0.001, ns, not significant (two-way ANOVA).

To assess the functional relevance of circZFYVE1 induction during IAV infection, both loss-of-function and gain-of-function experiments were conducted. Specific siRNAs targeting the unique back-splice junction of circZFYVE1 were designed. Transfection with these siRNAs resulted in an efficient reduction of circZFYVE1 expression compared to negative control siRNA (**Figure 2A**). Knockdown of circZFYVE1 increased the replication of both A/WSN/33 (H1N1) and A/Hong Kong/1/68 (H3N2), as determined by viral titers at 24 h post-infection (hpi) (**Figures 2B, C**). These results indicate that endogenous circZFYVE1 contributes to the restriction of IAV replication. To further assess the effect of circZFYVE1 gain of function, a circZFYVE1 overexpression plasmid, pcDNA-circZFYVE1, was constructed as previously described (25). This plasmid led to a robust increase in circZFYVE1 expression compared with control cells (**Figure 2D**). We note that this represents a strong ectopic overexpression condition, which does not precisely reflect the more modest induction observed during IAV infection. Therefore, the overexpression data should be interpreted primarily as evidence of antiviral potential and mechanistic directionality. Under this overexpression condition, circZFYVE1 did not significantly reduce viral titers at 12 hpi, although NP protein expression was decreased (**Figures 2E, F**). At 24 hpi, circZFYVE1 overexpression reduced both viral titers and NP protein levels for WSN (H1N1) and HK (H3N2) viruses (**Figures 2E, F**). Together with the loss-of-function results, these findings support a role for circZFYVE1 in restricting IAV replication, while indicating that the gain-of-function results should be interpreted in the context of strong ectopic overexpression.

### circZFYVE1 is a virus-induced host factor that functions independently of its parental gene

**Figure 3:**
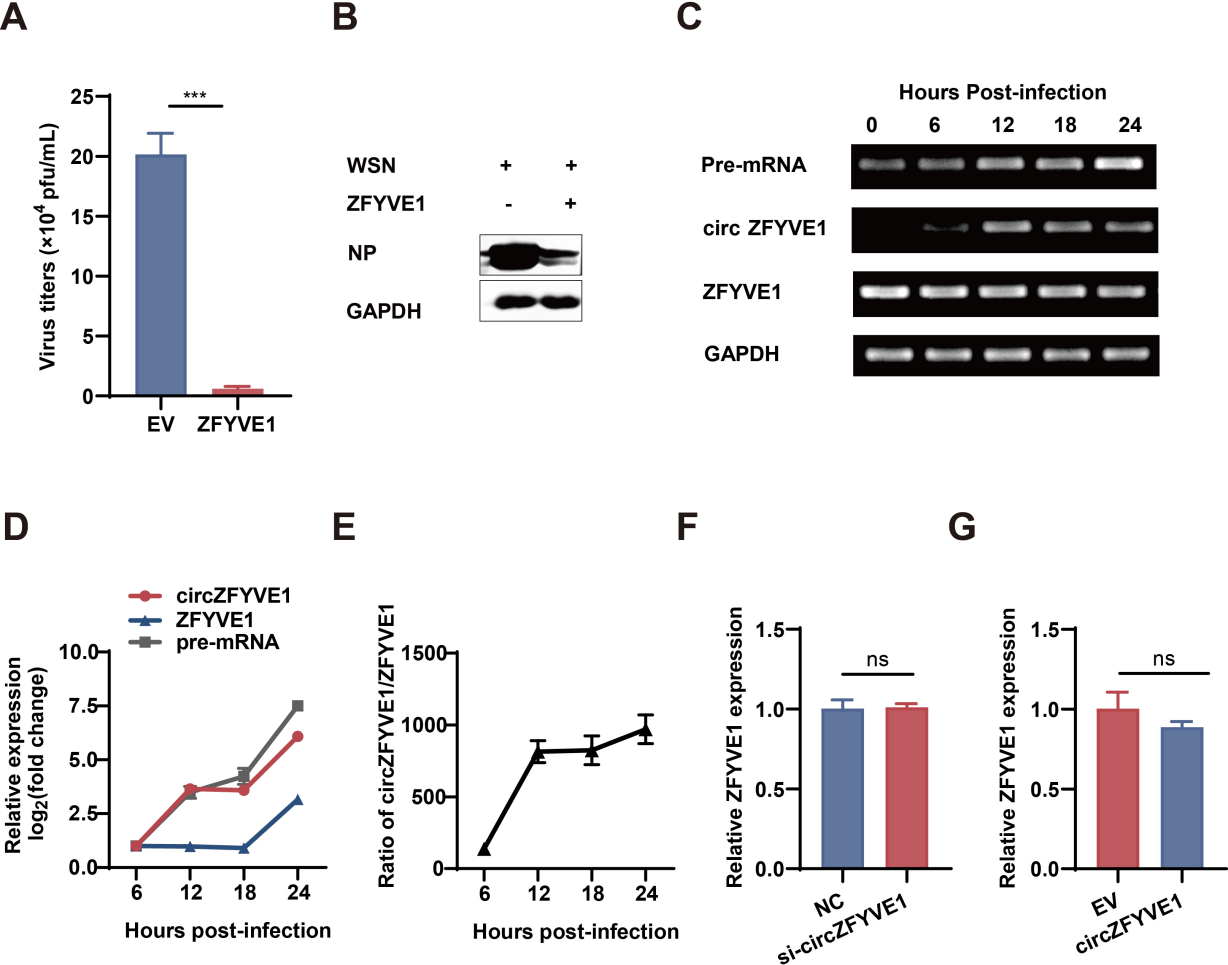
The antiviral function of circZFYVE1 is independent of its parental gene ZFYVE1. **(A, B)** The parental ZFYVE1 gene itself exhibits antiviral activity. **(A)** A549 cells overexpressing linear ZFYVE1 mRNA (ZFYVE1) or empty vector (EV) were infected with WSN virus at MOI of 0.001. Virus titers in the supernatant were measured at 24 hpi. **(B)** Western blot analysis of viral NP protein in cells described in **(A)** at 24 hpi. GAPDH serves as a loading control. Data in **(A, B)** are mean ± SD (n=3). *p < 0.05, **p < 0.01, ***p < 0.001, ns, not significant (Student’s t-test for A). **(C–E)** Expression dynamics of ZFYVE1-derived transcripts upon IAV infection. **(C)** IAV infection specifically upregulates ZFYVE1 pre-mRNA and circRNA, but not mature mRNA. Representative agarose gel of RT-PCR products for ZFYVE1 pre-mRNA, linear mRNA, and circRNA at the indicated times after WSN infection. **(D)** Expression kinetics of ZFYVE1-derived transcripts after IAV infection. A549 cells were infected with WSN (MOI = 1), and the levels of ZFYVE1 pre-mRNA, linear ZFYVE1 mRNA, and circZFYVE1 were analyzed by RT-qPCR at the indicated time points. Data are normalized to GAPDH and presented as mean ± SD (n=3). **(E)** The ratio of circZFYVE1 to linear ZFYVE1 mRNA in WSN-infected cells. **(F)** Cells were transfected with control (si-NC) or circZFYVE1-specific siRNA (si-circZFYVE1). The level of ZFYVE1 mRNA was quantified by qRT-PCR. Data are mean ± SD (n=3). *p < 0.05, **p < 0.01, ***p < 0.001, ns, not significant (Student’s t-test). **(G)** Cells were transfected with empty vector(EV) or circZFYVE1 overexpression plasmid(circZFYVE1). The level of ZFYVE1 mRNA was quantified by qRT-PCR. Data are mean ± SD (n=3). *p < 0.05, **p < 0.01, ***p < 0.001, ns, not significant (Student’s t-test).

The parental ZFYVE1 protein has been reported to enhance TLR3-mediated innate immune and inflammatory responses by facilitating ligand binding (28). We therefore assessed the antiviral potential of linear ZFYVE1 during IAV infection. In A549 cells, overexpression of ZFYVE1 resulted in a reduction in viral titers and NP protein levels at 24 hpi (**Figures 3A, B**), indicating that linear ZFYVE1 also has antiviral activity. Given the established immunological function of ZFYVE1 and the virus-induced accumulation of circZFYVE1, we next examined whether circZFYVE1 restricts viral replication through modulation of its parental gene expression. Analysis of ZFYVE1-derived transcripts revealed a linear-to-circular shift at the ZFYVE1 locus following IAV infection. Both ZFYVE1 pre-mRNA and circZFYVE1 levels increased over time, whereas the abundance of mature linear ZFYVE1 mRNA remained unchanged throughout the course of infection (**Figures 3C, D**). Consequently, the ratio of circZFYVE1 to linear ZFYVE1 mRNA increased markedly during infection, reaching a peak at 12 hpi (**Figure 3E**) and remaining elevated thereafter. This pattern suggests preferential production of the circular isoform relative to its linear counterpart in response to viral infection. Furthermore, neither siRNA-mediated knockdown nor overexpression of circZFYVE1 significantly altered the steady-state levels of linear ZFYVE1 mRNA (**Figures 3F, G**). Collectively, these results indicate that circZFYVE1 functions as a virus-responsive RNA species with regulatory activity distinct from that of the linear transcript.

### circZFYVE1 shows no detectable protein-coding activity under the tested conditions

**Figure 4:**
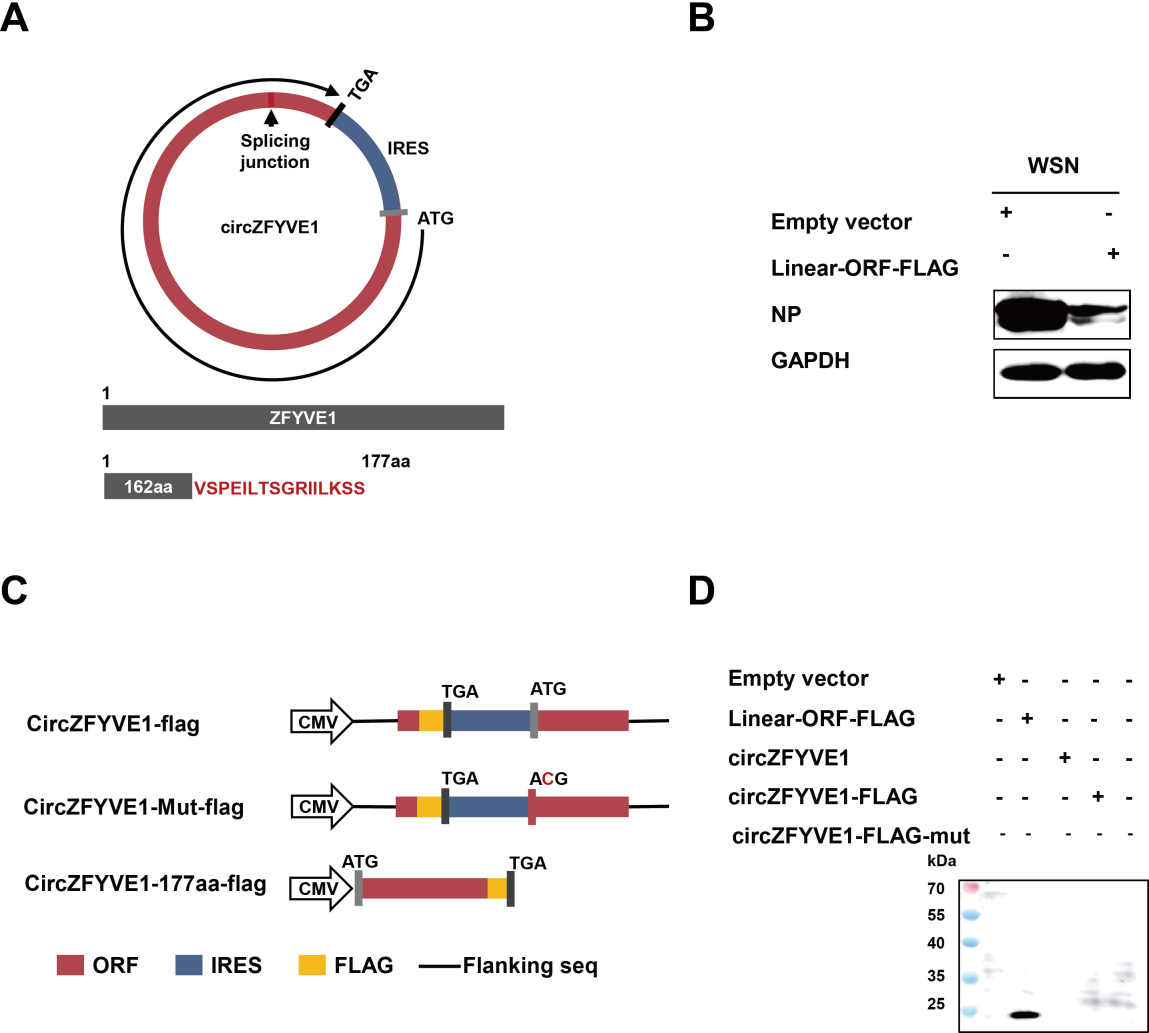
circZFYVE1 shows no detectable protein-coding activity under the tested conditions. **(A)** Diagram of the structure of the predicted ORF spanning the junction of circZFYVE1. **(B)** The putative ORF inhibits the replication of IAV in A549 cells. A549 cells were transfected with pcDNA-linear-177aa-orf (Linear-ORF-FLAG) and empty vector (EV) respectively, and 24 h later, the cells were infected with WSN at MOI of 0.01. Cell lysates were collected and used for Western blot with anti-IAV NP protein monoclonal antibody. **(C)** Schematic diagram of circZFYVE1-Flag and Mut plasmid construction. **(D)** Detection of the products from the predicted ORF and control plasmid using anti-FLAG antibodies.

Although circRNAs are generally considered non-coding, accumulating evidence suggests that some circRNAs can be translated through internal open reading frames (ORFs) (29, 30). Sequence analysis identified a putative linear ORF within circZFYVE1 predicted to encode a 177-amino acid protein, termed circZFYVE1-177aa, including a unique 16-amino acid sequence spanning the back-splice junction (**Figure 4A**). Given that the parental ZFYVE1 protein enhances TLR3-mediated innate immune responses (28), the potential antiviral activity of the predicted ORF was subsequently examined. The full-length linear ORF of circZFYVE1 was cloned into the pcDNA3.1 expression vector. Overexpression of this construct reduced viral replication at 24 hpi (**Figure 4B**), indicating antiviral activity of the encoded protein. To determine whether circZFYVE1 itself produces the predicted protein, a circZFYVE1 expression plasmid containing flanking intronic sequences required for circularization was generated (**Figure 4C**). A C-terminal FLAG tag was introduced into the predicted ORF to facilitate detection, and a linear 177aa-FLAG construct served as a positive control. Immunoblot analysis detected a FLAG-tagged protein (~25 kDa) in cells expressing the linear ORF construct, whereas no corresponding signal was observed in cells expressing circZFYVE1 (**Figure 4D**). These findings indicate that, although the linear ORF can be translated and displays antiviral activity, circZFYVE1 does not produce the predicted protein under the experimental conditions tested. Therefore, circZFYVE1 is unlikely to exert its antiviral activity through protein coding, prompting us to examine whether it functions through a non-coding regulatory mechanism, such as acting as a ceRNA.

### circZFYVE1 functions as a miRNA sponge for miR-4435

**Figure 5:**
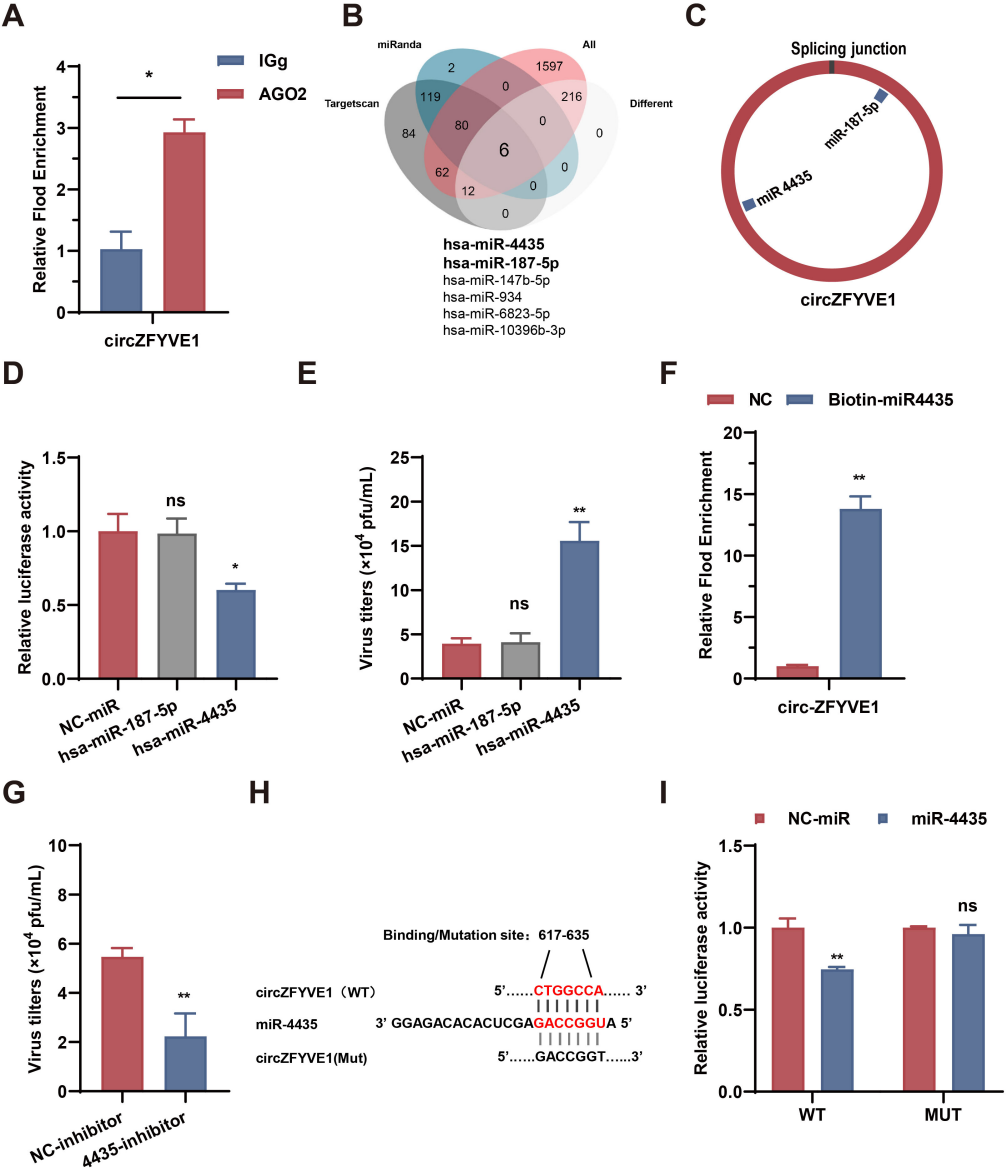
circZFYVE1 functions as a miRNA sponge for miR-4435. **(A)** RNA immunoprecipitation (RIP) analysis of circZFYVE1 association with Ago2. A549 cells were transfected with Ago2-Flag or control vector, followed by immunoprecipitation using anti-Flag antibody. Enrichment of circZFYVE1 was quantified by qRT-PCR and compared with IgG control. Data are mean ± SD (n=3). *p < 0.05, **p < 0.01, ***p < 0.001, ns, not significant (Student’s t-test). **(B)** Venn diagram showing the overlap of candidate miRNAs predicted to bind circZFYVE1 using TargetScan, miRanda, and microRNA-seq data. The pink represents the whole detected microRNA, the white represents the dysregulated microRNA. Two candidate miRNAs were selected for further validation. **(C)** Schematic drawing showing the putative binding sites of the miRNAs associated with circZFYVE1. **(D)** Luciferase reporter assays showing the interaction between circZFYVE1 and candidate miRNAs. The circZFYVE1 sequence was cloned downstream of the luciferase reporter and co-transfected into 293T cells with negative control miRNA (NC-miR), hsa-miR-4435, or hsa-miR-187-5p mimics. Relative luciferase activity was measured and normalized to the control group. Data are mean ± SD (n=3). *p < 0.05, **p < 0.01, ***p < 0.001, ns, not significant. **(E)** Viral titers of WSN in A549 cells transfected with miR-4435 mimics, hsa-miR-187-5p mimics, or NC-miR, followed by infection at an MOI of 0.01. Viral titers in the supernatants were determined at 24 hpi. Data are mean ± SD (n=3). *p < 0.05, **p < 0.01, ***p < 0.001, ns, not significant. **(F)** RNA pull-down assays using biotin-labeled miR-4435 or negative control miRNA (NC). Enrichment of circZFYVE1 was analyzed by qRT-PCR and compared with NC control. Data are mean ± SD (n=3). *p < 0.05, **p < 0.01, ***p < 0.001, ns, not significant (Student’s t-test). **(G)** Viral titers of WSN in A549 cells transfected with miR-4435 inhibitors, NC-inhibitors, followed by infection at MOI of 0.01. Viral titers in the supernatants were determined at 24 hpi. Data are mean ± SD (n=3). *p < 0.05, **p < 0.01, ***p < 0.001, ns, not significant (Student’s t-test). **(H)** Schematic representation of the predicted miR-4435 binding site within circZFYVE1 and the corresponding mutant construct used for luciferase reporter assays. **(I)** Luciferase reporter assays performed in 293T cells co-transfected with wild-type (WT) or mutant (MUT) circZFYVE1 reporter constructs and miR-4435 mimics or NC-miR. Relative luciferase activity was measured and normalized to the control group. Data are mean ± SD (n=3). *p < 0.05, **p < 0.01, ***p < 0.001, ns, not significant (two-way ANOVA).

Given the predominant cytoplasmic localization of circZFYVE1, we next examined whether circZFYVE1 regulates viral replication through a ceRNA mechanism by interacting with miRNAs. Because miRNAs exert their function through association with Argonaute 2 (Ago2), a core component of the RNA-induced silencing complex (RISC) (31), and circZFYVE1 is predominantly localized in the cytoplasm, we assessed whether circZFYVE1 associates with Ago2. A549 cells were transfected with Ago2-flag or control vector (pcDNA3.1-flag) and RNA immunoprecipitation (RIP) assays demonstrated that circZFYVE1 was enriched in Ago2-FLAG immunoprecipitates compared with control samples (**Figure 5A**), suggesting its association with the RISC complex. Next, to identify specific miRNAs that interact with circZFYVE1, bioinformatics prediction tools such as TargetScan and miRanda were used. Based on the predictions, two candidate miRNAs, hsa-miR-4435 and hsa-miR-187-5p, were selected for further validation (**Figures 5B, C**). To validate these predictions, the linear sequence of circZFYVE1 was cloned into a luciferase reporter vector and co-transfected with miRNA mimics in 293T cells followed by luciferase activity measurement. Luciferase activity was reduced in the presence of hsa-miR-4435, whereas no significant change was observed with hsa-miR-187-5p (**Figure 5D**). RNA pull-down assays using biotin-labeled miR-4435 further confirmed enrichment of circZFYVE1 compared with a negative control miRNA. The results (**Figure 5F**) revealed that circZFYVE1 could be efficiently enriched by biotin-labeled miR-4435, but not by the negative control miRNA (NC-miR), supporting a direct interaction. Functional analysis showed that miR-4435 overexpression increased viral titers, whereas miR-4435 inhibition reduced viral replication in IAV-infected A549 cells (**Figures 5E, G**), supporting the role of miR-4435 in promoting IAV replication. To determine whether the predicted miR-4435 binding site in circZFYVE1 sequence was essential for their interaction, the predicted miR-4435 binding site in circZFYVE1 was inserted into a luciferase reporter vector (**Figure 5H**). A decrease in luciferase activity was observed in cells transfected with the wild-type construct, but no significant reduction was detected when the binding site was mutated (**Figure 5I**), indicating that this site is required for the interaction. Collectively, these results support that circZFYVE1 functions as a sponge for miR-4435, thereby modulating IAV replication.

### circZFYVE1 functions as a ceRNA for miR-4435 to modulate LSM14A-mediated antiviral responses

**Figure 6:**
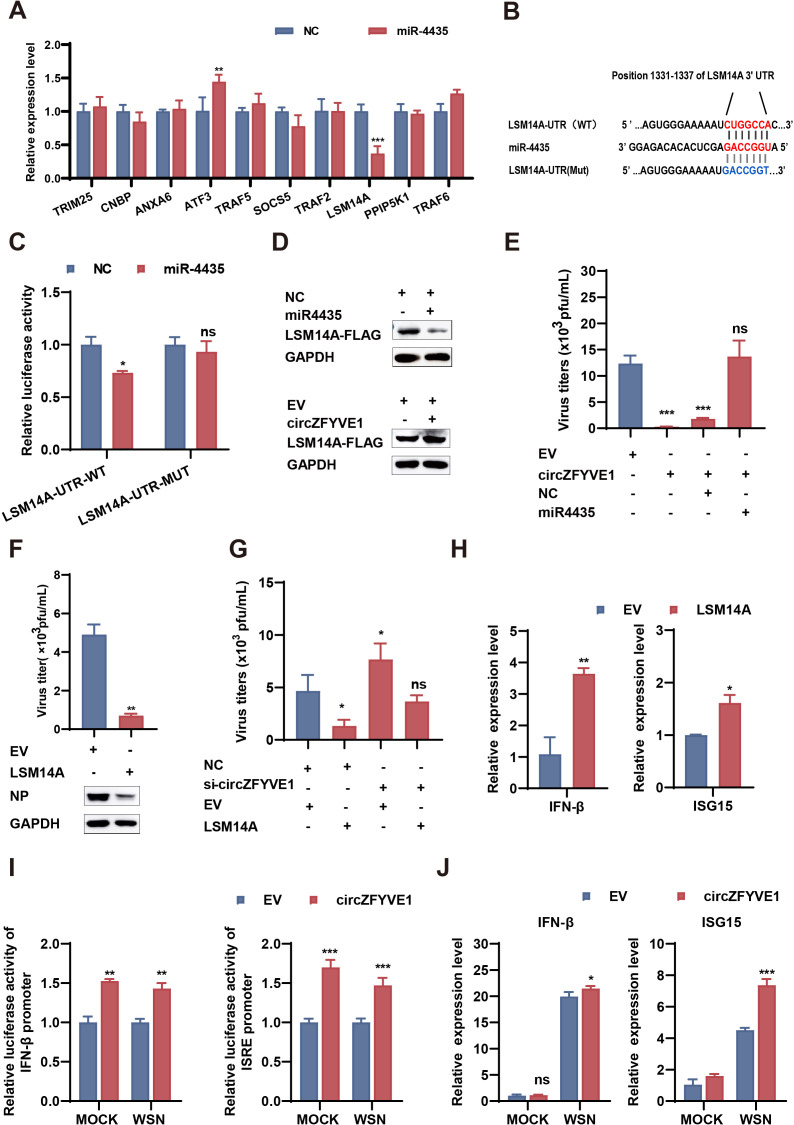
The circZFYVE1 functions as a ceRNA for miR-4435 to modulate LSM14A-mediated antiviral responses. **(A)** Relative mRNA levels of the candidate target genes of miR-4435 were evaluated by qRT-PCR in A549 cells transfected with the miR-4435 mimics. Data are normalized to GAPDH and presented as mean ± SD (n=3), *p < 0.05, **p < 0.01, ***p < 0.001, ns, not significant. **(B)** Schematic representation of the predicted miR-4435 binding site within 3’UTR of LSM14A and the corresponding mutant construct used for luciferase reporter assays. **(C)** Luciferase reporter assays performed in 293T cells co-transfected with wild-type (WT) or mutant (MUT) LSM14A 3’UTR reporter constructs and miR-4435 mimics or NC-miR. Relative luciferase activity was measured and normalized to the control group. Data are mean ± SD (n=3). *p < 0.05, **p < 0.01, ***p < 0.001, ns, not significant (two-way ANOVA). **(D)** CircZFYVE1 and miR-4435 regulate the expression of a FLAG-tagged LSM14A-3’ UTR reporter construct. 293T cells were co-transfected with pCMV-LSM14A-3’ UTR-FLAG together with miR-4435 mimics or the circZFYVE1 expression plasmid, respectively. The expression of the FLAG-tagged LSM14A-3’ UTR reporter was detected by western blotting using an anti-FLAG antibody. **(E)** Viral titers in the supernatants of A549 cells co-transfected with circZFYVE1 and miR-4435 mimics followed by IAV infection (WSN, MOI = 0.01). A549 cells were transfected with the indicated miRNA mimics or the indicated plasmids for 24 h and then infected with WSN. The supernatants of infected cells were collected 24 h post-infection for plaque tests to determine virus titer. Data are mean ± SD (n=3). *p < 0.05, **p < 0.01, ***p < 0.001, ns, not significant. **(F)** LSM14A inhibits IAV replication in A549 cells. A549 cells were transfected with the LSM14A expression plasmid or empty vector for 24 h and then infected with WSN at an MOI of 0.01. Supernatants were collected at 24 h post-infection for plaque assay, and cell lysates were analyzed by western blotting using an anti-IAV NP antibody. GAPDH served as a loading control. Data are presented as mean ± SD (n = 3). *p < 0.05, **p < 0.01, ***p < 0.001, ns, not significant. **(G)** Restoration of LSM14A expression rescues the increased viral replication caused by circZFYVE1 knockdown. A549 cells were transfected with si-circZFYVE1 or control siRNA, followed by transfection with the LSM14A expression plasmid or empty vector. Cells were then infected with WSN at an MOI of 0.01. Supernatants were collected at 24 h post-infection, and viral titers were determined by plaque assay. Data are presented as mean ± SD (n = 3). *p < 0.05, **p < 0.01, ***p < 0.001, ns, not significant. **(H)** LSM14A increased IFN-β and ISG15 gene expression. The A549 cells were transfected with LSM14A plasmid for 24 h and then qRT-PCR analysis for IFN-β and ISG15 mRNA was performed. Data are normalized to GAPDH and presented as mean ± SD (n=3), *p < 0.05, **p < 0.01, ***p < 0.001, ns, not significant. **(I)** CircZFYVE1 activated IFN-β and the ISRE promoter. The A549 cells were transfected with IFN-β or ISRE reporter and circZFYVE1 plasmid for 24 h, and then left uninfected or infected with WSN for 12 h before luciferase assays. **(J)** CircZFYVE1 increased IFN-β and ISG15 gene expression. The A549 cells were transfected with circZFYVE1 plasmid for 24 h and then uninfected or infected with WSN for 12 h, qRT-PCR analysis for IFN-β and ISG15 mRNA was performed. Data are normalized to GAPDH and presented as mean ± SD (n=3), *p < 0.05, **p < 0.01, ***p < 0.001, ns, not significant.

MicroRNAs regulate gene expression by binding to complementary sequences within the 3’ untranslated region (UTR) of target mRNAs. To identify potential downstream targets of miR-4435, candidate genes were predicted using TargetScan and miRanda. Based on these predictions, together with their reported or annotated involvement in innate immune or antiviral signaling pathways and detectable expression in A549 cells, ten candidate miR-4435 target genes were selected for validation. A549 cells were transfected with miR-4435 mimics or negative control mimics, and the mRNA levels of these candidate genes were examined by qRT-PCR. Among the tested genes, LSM14A mRNA was markedly reduced upon miR-4435 overexpression compared with the negative control group (**Figure 6A**).

To determine whether LSM14A is directly targeted by miR-4435, the wild-type LSM14A 3′UTR containing the predicted miR-4435-binding site or a mutant construct with substitutions in the seed-matching region was cloned into the pmirGLO luciferase reporter vector (**Figure 6B**). Dual-luciferase assays showed that miR-4435 reduced luciferase activity of the WT construct, whereas this effect was abolished in the MUT construct (**Figure 6C**), supporting a direct 3′UTR-dependent regulatory interaction. To further assess whether miR-4435 and circZFYVE1 regulate LSM14A through its 3′UTR, we used a FLAG-tagged LSM14A-3′UTR reporter construct. Western blot analysis using an anti-FLAG antibody showed that miR-4435 overexpression reduced reporter expression, whereas circZFYVE1 overexpression increased reporter expression under the tested conditions (**Figure 6D**). These data support a 3’ UTR-dependent regulatory relationship between miR-4435/circZFYVE1 and LSM14A. We next examined whether the antiviral effect of circZFYVE1 depends on miR-4435. Overexpression of circZFYVE1 reduced IAV WSN replication in A549 cells, whereas co-transfection with miR-4435 mimics partially reversed this inhibitory effect (**Figure 6E**). These results suggest that circZFYVE1 restricts IAV replication, at least in part, by modulating miR-4435 activity. Given the reported role of LSM14A in antiviral signaling, we further evaluated its functional contribution during IAV infection. Ectopic expression of LSM14A reduced viral titers and decreased viral NP protein levels in WSN-infected A549 cells (**Figure 6F**). To determine whether LSM14A functionally mediates the antiviral effect of circZFYVE1, we performed a rescue experiment in circZFYVE1-depleted A549 cells. Knockdown of circZFYVE1 increased WSN replication, whereas ectopic expression of LSM14A significantly reduced viral titers in circZFYVE1-depleted cells (**Figure 6G**). These results indicate that restoration of LSM14A expression can partially rescue the impaired antiviral phenotype caused by circZFYVE1 knockdown, supporting LSM14A as a functionally relevant downstream mediator of circZFYVE1. In addition, LSM14A overexpression enhanced the mRNA expression of IFN-β and ISG15 (**Figure 6H**), supporting its role in promoting antiviral responses.

Finally, we assessed the effect of circZFYVE1 on antiviral innate immune signaling. circZFYVE1 overexpression increased both IFN-β and ISRE promoter reporter activities under mock and WSN-infected conditions (**Figure 6I**). Consistently, circZFYVE1 enhanced the expression of IFN-β and ISG15 during WSN infection, with a more pronounced effect on ISG15 expression (**Figure 6J**). Together, these results support a regulatory model in which circZFYVE1 functions as a ceRNA for miR-4435 to relieve miR-4435-mediated repression of LSM14A, thereby enhancing antiviral innate immune responses and restricting IAV replication, at least in part through the miR-4435/LSM14A axis.

## Discussion

In this study, we identified circZFYVE1 as an IAV-inducible circular RNA that modulates host innate immune responses to viral infection. Our data indicate that IAV infection alters transcript output from the ZFYVE1 locus, with preferential accumulation of circZFYVE1 while mature linear ZFYVE1 mRNA remains relatively stable. Mechanistically, our findings support a model in which circZFYVE1 acts, at least in part, as a ceRNA for miR-4435, thereby relieving miR-4435-mediated repression of LSM14A and enhancing downstream antiviral signaling.

The mechanisms underlying circRNA regulation during infection remain largely unknown. Here, we show that IAV infection is associated with differential accumulation of ZFYVE1-derived linear and circular transcripts. Following IAV infection, host cells maintain stable steady-state levels of mature linear ZFYVE1 mRNA, whereas ZFYVE1 pre-mRNA and circZFYVE1 exhibit time-dependent accumulation. This differential expression pattern suggests that circZFYVE1 is not merely a passive transcriptional byproduct, but rather may have an independent regulatory role during viral infection. Importantly, circZFYVE1 modulates IAV replication without detectably altering the steady-state mRNA abundance of its parental gene. Nevertheless, given the emerging role of circRNAs as molecular scaffolds (32), it remains plausible that circZFYVE1 could influence ZFYVE1 at the post-translational level, for example, by affecting ZFYVE1 protein stability or virus-induced protein turnover—a possibility that was not directly examined in the present study and warrants further investigation.

Recent studies have demonstrated that certain circRNAs containing ORFs can be translated into functional peptides via cap-independent mechanisms involving internal ribosome entry sites (IRES) or N6-methyladenosine (m6A) modifications (33–35). For instance, Zheng et al. identified circYthdc2 in lower vertebrate fish, which can translate into a 170 amino acid polypeptide (Ythdc2-170aa) through an IRES sequence or m6A modification, playing a role in antiviral immunity (36). Bioinformatic analyses revealed that circZFYVE1 also possesses an ORF and an IRES. However, under the conditions tested, circZFYVE1 did not produce a detectable peptide, even though its linear ORF could be translated. This finding supports the view that circZFYVE1 functions predominantly as a non-coding RNA under our experimental conditions and is consistent with a model in which its biological impact is mediated mainly through RNA-based molecular interactions, particularly the ceRNA mechanism. In addition to IRES-dependent translation, m6A-dependent regulation remains an important possibility. m6A modification has been reported to influence circRNA stability, localization, immune recognition, and, in some cases, cap-independent translation. Although our current data did not support detectable protein-coding potential for circZFYVE1 under the tested conditions, we cannot exclude the possibility that m6A modification may affect the stability, subcellular distribution, or regulatory activity of circZFYVE1 during IAV infection. Future studies using m6A-RIP-qPCR, site-specific mutagenesis, or perturbation of m6A writers/readers will be useful for determining whether m6A-dependent regulation contributes to circZFYVE1 biology.

Exonic circRNAs are predominantly cytoplasmic and frequently act as miRNA sponges (31, 37). Multiple studies have delineated circRNA–miRNA–mRNA regulatory networks that modulate immune responses during infections with various viruses, including Zika virus, Seneca virus A, influenza A virus, and coxsackievirus B3 (21, 38–40). Building on these paradigms, we hypothesized that circZFYVE1 modulates IAV replication through a similar post-transcriptional mechanism. Using miRNA target prediction tools, including TargetScan and miRanda, combined with miRNA sequencing data, we identified and validated miR-4435 as a direct target of circZFYVE1 through biotin-coupled RNA pull-down and luciferase reporter assays. These data support a ceRNA-like mechanism in which circZFYVE1 interacts with miR-4435 and attenuates its inhibitory effect on LSM14A. By modulating miR-4435 activity, circZFYVE1 may help maintain LSM14A availability during infection, thereby supporting antiviral signaling, including IFN-β and ISG15 induction. However, ceRNA activity is highly dependent on the relative abundance of the circRNA, the miRNA, and the target mRNA. In this study, IAV infection induced endogenous circZFYVE1 by approximately 20- to 30-fold, whereas plasmid-mediated overexpression resulted in a much higher level of circZFYVE1 expression. Therefore, the gain-of-function experiments should be interpreted primarily as evidence for the antiviral potential and mechanistic directionality of circZFYVE1, rather than as a precise mimic of its physiological induction level during IAV infection. In addition, the circZFYVE1/linear ZFYVE1 ratios shown in **Figure 3C–E** reflect relative changes in transcript abundance, but do not provide absolute copy numbers. Future studies using endogenous-level rescue systems and absolute quantification of circZFYVE1, miR-4435, and LSM14A transcripts will be required to determine whether infection-induced circZFYVE1 is stoichiometrically sufficient to sponge endogenous miR-4435 under physiological infection conditions.

Our analysis further identified LSM14A as a direct and functionally relevant downstream target of miR-4435. The ten candidate miR-4435 target genes examined in the initial screen were selected based on bioinformatic prediction using TargetScan and miRanda, the presence of predicted miR-4435-binding sites within their 3′ UTRs, their annotated or reported relevance to innate immune or antiviral signaling pathways, and detectable expression in A549 cells. Among these candidates, LSM14A mRNA showed a clear response to miR-4435 modulation and was further validated by luciferase reporter assays and functional analyses. Nevertheless, the rescue effect observed in our functional assays was partial, indicating that circZFYVE1-mediated antiviral activity may not be exclusively dependent on the miR-4435/LSM14A pathway. Other miR-4435 targets, as well as potential miR-4435-independent mechanisms, may also contribute to the overall antiviral phenotype. We did not systematically examine all reported or predicted antiviral targets of miR-4435, and therefore additional miR-4435-regulated genes may also participate in circZFYVE1-mediated restriction of IAV replication. Transcriptome-wide and proteome-wide analyses will be valuable for defining the broader miR-4435-regulated antiviral network during IAV infection.

From an evolutionary perspective, virus-induced circZFYVE1 accumulation may represent a compensatory host defense program. IAV NS1 protein is known to antagonize host antiviral defenses at multiple levels (41, 42), including suppression of RIG-I/IRF3-associated signaling and modulation of RNA granule-related pathways. Given the reported involvement of LSM14A/RAP55 in P-bodies, stress granules, and antiviral signaling (24),it is tempting to speculate that circZFYVE1-mediated regulation of LSM14A may intersect with NS1-dependent immune evasion. However, this possibility remains speculative, as the present study did not directly examine NS1 binding, NS1-dependent antagonism, or RNA granule dynamics. Future studies using NS1 mutants, co-localization analyses with P-body or stress granule markers, and interaction assays involving NS1, LSM14A, and circZFYVE1 will be required to determine whether and how the circZFYVE1/LSM14A axis intersects with NS1-mediated modulation of RNA granule-associated antiviral signaling. Collectively, these findings broaden our understanding of circRNA-mediated immune regulation and highlight the circZFYVE1/miR-4435/LSM14A axis as a host regulatory pathway involved in antiviral defense.

The identification of this endogenous regulatory circuit may also have implications for the development of host-directed antiviral strategies, although such translational potential should be interpreted cautiously. Current anti-influenza strategies, which primarily target viral proteins, face ongoing challenges due to rapid viral mutation and the emergence of drug-resistant strains. Host-directed approaches that enhance innate antiviral immunity may therefore provide complementary strategies. However, the present study was performed mainly in immortalized cell lines, and the antiviral activity, delivery feasibility, safety, and immunogenicity of circZFYVE1-based or miR-4435-targeted interventions remain to be evaluated. Thus, rather than establishing circZFYVE1 as an immediate therapeutic candidate, our findings suggest that the circZFYVE1/miR-4435/LSM14A axis represents a potential target for further investigation in primary airway epithelial cells, differentiated airway cultures, and *in vivo* infection models.

In summary, we identify circZFYVE1 as a previously unrecognized, IAV-inducible circRNA that modulates host innate immune responses to viral infection. Functionally, circZFYVE1 restricts IAV replication at least in part through a ceRNA-like mechanism involving miR-4435 and LSM14A, thereby supporting antiviral signaling. These findings highlight the functional importance of circRNA–miRNA–mRNA regulatory networks in host–pathogen interactions and suggest that the circZFYVE1/miR-4435/LSM14A axis may serve as a potential target for future studies of host-directed antiviral responses.

## Data Availability

The original contributions presented in the study are included in the article/**Supplementary Material**. Further inquiries can be directed to the corresponding author.
