# Supplementary figures and images for: CircZFYVE1 functions as a competitive endogenous RNA to enhance LSM14A-mediated antiviral defense against influenza A virus
Source: Front Immunol. 2026 Jul 9;17:1841925. doi: 10.3389/fimmu.2026.1841925 (PMC13391820; doi:10.3389/fimmu.2026.1841925)

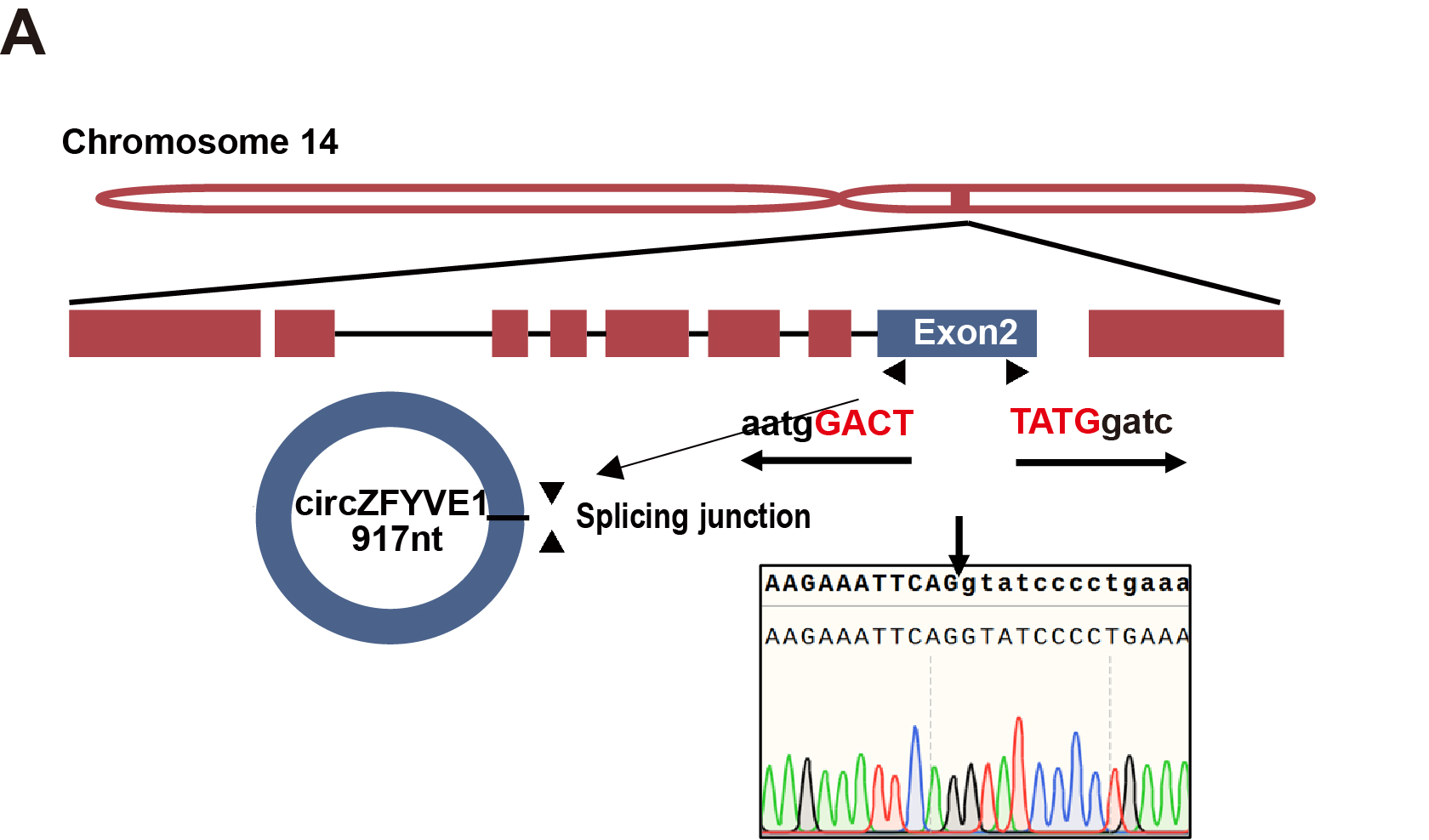

Supplement: Supplementary file 1 [file Image1.jpeg]
