# Supplementary material for: CircZFYVE1 functions as a competitive endogenous RNA to enhance LSM14A-mediated antiviral defense against influenza A virus
Source: Front Immunol. 2026 Jul 9;17:1841925. doi: 10.3389/fimmu.2026.1841925 (PMC13391820; doi:10.3389/fimmu.2026.1841925)
Supplement: Supplementary file 2 [file Table1.docx]

Supplementary Table 1. Sequences of primers, siRNA, miRNA, and miRNA inhibitors used in this study.

| Purpose | Primer | Sequence (5'-3') |
| --- | --- | --- |
| siRNA | circZFYVE1-Si-1 | GAAGAAAUUCAGGUAUCCCTT |
|  | circZFYVE1-Si-2 | AGAAAUUCAGGUAUCCCCUTT |
| qPCR | GAPDH--F | ACAACTTTGGTATCGTGGAAGG |
|  | GAPDH- -R | GCCATCACGCCACAGTTTC |
|  | circZFYVE1-F | GACGAAAATGAAGAAATTCAGGTA |
|  | circZFYVE1-R | TGATGGGTTCACGGTGGT |
|  | circZFYVE1-pre-mRNA-F | AGCCTACTTCATCGAGTTGTGA |
|  | circZFYVE1-pre-mRNA-R | CATTTACTCACAGCTTGCCAG |
|  | ZFYVE1-F | TGGTGCGGACAGAGATTGTG |
|  | ZFYVE1-R | GACACCGACTGAGCCATGAA |
|  | IFNβ-F | GCTTGGATTCCTACAAAGAAGCA |
|  | IFNβ-R | ATAGATGGTCAATGCGGCGTC |
|  | ISG15-F | CGCAGATCACCCAGAAGATCG |
|  | ISG15-R | TTCGTCGCATTTGTCCACCA |
|  | TRIM25_F | AGCAGCTACAACAAGAATACACG |
|  | TRIM25_R | GGCTCTGTTCAATCTCCTCCT |
|  | CNBP-F | GGAGCCCAAGAGAGAGCGA |
|  | CNBP-R | TGGCTACATGACCAGTTTCAC |
|  | ANXA6_F | ACGGTTGATTGTGGGCCTG |
|  | ANXA6_R | GTGCATCTGCTCATTGGTCC |
|  | TRAF5_F | CCACTCGGTGCTTCACAAC |
|  | TRAF5_R | GTACCGGCCCAGAATAACCT |
|  | SOCS5-F | AGAGATTCCTACTCTCGACATGC |
|  | SOCS5-R | GCCCACAGTATCCTGCAACC |
|  | TRAF2_F | GCTCATGCTGACCGAATGTC |
|  | TRAF2_R | GCCGTCACAAGTTAAGGGGAA |
|  | PPIP5K1-F | TCCGAAAGACGGGGTCGTA |
|  | PPIP5K1-R | TGTCTCGTTCAACCTTCCCAT |
|  | TRAF6-F | ATGCGGCCATAGGTTCTGC |
|  | TRAF6-R | TCCTCAAGATGTCTCAGTTCCAT |
|  | LSM14A-F | CTTATGGACCTTTCGGCAGGA |
|  | LSM14A-R | GGCAGAACCAACCGCACTA |
| CircZFYVE1 overexpression plasmid (pcDNA- CircZFYVE1) construction | Linear circZFYVE1 with flanking sequence-F | CAAAATCTAAGTCCAGATATCGA  GCCGAGACTGTCCCATTG |
|  | Linear circZFYVE1 with flanking sequence-R | ATCTAAGTCCAAGGCTCAGAAA  AGTAAAGTAATTCGG |
|  | Downstream-F | TCTGAGCCTTGGACTTAGATT  TTGTTCCTAAACAGA |
|  | Downstream-R | AACGGGCCCTCTAGACTCGAGTTTCT  TAATCATCTGAAGCATGGAG |
|  | Upstream-F | AGTCCAGTGTGGTGGAATT  CTTTCTTAATCATCTG |
|  | Upstream-R | GCCGCCACTGTGCTGGATATC  TGGACTTAGATTTTG |
| miR mimics& inhibitor | hsa-miR-187-5p | GGCUACAACACAGGACCCGGGC |
|  | hsa-miR-4435 mimics | AUGGCCAGAGCUCACACAGAGG |
|  | hsa-miR-4435 inhibitor | CCUCUGUGUGAGCUCUGGCCAU |
| circZFYVE1 luciferase reporter plasmid (pmirGLO- circZFYVE1-WT) construction GLO- | GLO-WT-F | GTTGTTTAAACGAGCTCGCTA  GCGTATCCCCTGAAATAC |
|  | GLO-WT-R | TGCCTGCAGGTCGACTCTAG  ACTGAATTTCTTCATTTTCG |
| circZFYVE1 luciferase reporter plasmid (pmirGLO- circZFYVE1-MUT) construction GLO- | GLO-MUT-F | AGCGGATAAGACTCAAACGA  CCGGTTGTCCCTTACTGTGACCTC |
|  | GLO-MUT-R | TGCCTGCAGGTCGACTCTA  GACTGAATTTCTTCATTTTCG |
| LSM14A expression vector | PCA- LSM14A -CDS-F | gacgacgatgacaaggaattcATGAGCGGGGGCACCCCTTACATC |
|  | PCA- LSM14A -CDS-R | aaaaagatctgctagctcgagCTATGCAGCAACTTTGTTGTC |
| The LSM14A expression reporter vectors | pcmv-LSM14A-3223-F | aaggacgacgatgacaagcttATGAGCGGGGGCACCCCTTACATC |
|  | pcmv- LSM14A-3223-R | agatctcggtcgaccgaattcAGGGCTGGGAAAACGCATAAACTC |
| LSM14A luciferase reporter plasmid construction (pmirGLO LSM14A -UTR-WT) | LSM14A -UTR-WT-F | gttgtttaaacgagctcgctagcAGTTTTCCAGGCTTAAC |
|  | LSM14A -UTR-WT -R | TGcctgcaggtcgactctagaAGGGCTGGGAAAACGCATAAAC |
| LSM14A luciferase reporter plasmid construction (pmirGLO LSM14A -UTR-MUT) | LSM14A -UTR- MUT -F | TAGTGGGAAAAATgaccggtCTTTTGTGTTTTTATGAAGGC |
|  | LSM14A -UTR- MUT-R | TAAAAACACAAAAGaccggtcATTTTTCCCACTAGAGC |
